# *Proximity Labeling*: Precise Proteomics Technology for Mapping Receptor Protein Neighborhoods at the Cancer Cell Surface

**DOI:** 10.3390/cancers17020179

**Published:** 2025-01-08

**Authors:** Saman Rahmati, Andrew Emili

**Affiliations:** Department of Biomedical Engineering, Division of Oncological Sciences, Knight Cancer Institute, Oregon Health & Science University, Portland, OR 97201, USA; rahmatis@ohsu.edu

**Keywords:** proximity labeling, cancer cell, receptor, interaction, BioID/TurboID, APEX, photocatalyst, affinity enrichment, mass spectrometry, spatial proteomics

## Abstract

Cell surface receptors are pivotal to cancer cell transformation, disease progression, metastasis, early detection, targeted therapy, drug responses, and clinical outcomes. Since they coordinate complex signaling communication networks in the tumor microenvironment, mapping the physical interaction partners of cell surface receptors in vivo is vital for understanding their roles, functional states, and suitability as therapeutic targets. Yet traditional methods like immunoprecipitation and affinity purification–mass spectrometry often fail to detect key but weak or transient receptor–protein interactions. Proximity labeling, a cutting-edge proteomics technology, addresses these technical challenges by enabling precise mapping of protein neighborhoods around a receptor target on the cell surface of cancer cells. This technique has been successfully applied in vitro and in vivo for proteomic mapping across various model systems. This review explores the fundamental principles, technologies, advantages, limitations, and applications of proximity labeling in cancer biology, focusing on mapping receptor microenvironments. By advancing mechanistic insights into cancer cell receptor signaling mechanisms, proximity labeling is poised to transform cancer research, improve targeted therapies, and illuminate avenues to overcome drug resistance.

## Simple Summary

Cell surface proteins and receptors on cancer cells, important as disease markers and drug targets, are highly dynamic, with their biological functions controlled by constantly changing protein–protein interaction networks. Studying these transient macromolecular neighborhoods is technically challenging, and traditional methods often fall short. Proximity labeling, a cutting-edge proteomics technology, offers a more precise approach that overcomes the limitations of existing methods. This review highlights the strategies, applications, benefits, and limitations of different proximity labeling platforms used to map the receptor protein microenvironment on the surface of cancer cells.

## 1. Introduction

Cell surface receptors are key drivers of oncogenesis, neoplastic progression, and metastatic dissemination, and so provide valuable therapeutic targets in oncology due to their critical roles in tumor cell biology. Many cancers overexpress specific receptors, and their extracellular location enables precise targeting by highly selective therapeutic agents, including antibodies. Targeting cell surface receptors offers key advantages, including the ability to disrupt dysregulated pathways driven by oncoproteins such as EGFR, HER2, VEGFR, and PD-PDL1, which are essential for cancer cell growth and survival [1,2,3,4,5]. For example, HER2 is overexpressed in 20–25% of breast cancers and can be targeted using antibodies such as trastuzumab that have significantly improved outcomes for these patients [6,7,8].

Combining agents to target multiple cell surface receptors further enhances selectivity, reducing toxicity and minimizing the risk of therapy resistance [4,9,10]. Beyond directly targeting cancer cell-intrinsic functions, receptor-based therapies can disrupt interactions between cancer cells and other components of the tumor microenvironment, such as immune cells, to facilitate therapeutic clearance. Yet cancer cells frequently show adaptive changes to therapy through the irregular expression and altered function of cell surface receptors in response to therapy, leading to abnormal behavior and eventual resistance to treatments. Hence, understanding the networks of protein–protein interactions (PPIs) within the dynamic cell surface microenvironment surrounding a cancer cell surface receptor in different neoplastic contexts is crucial to deciphering the complex biochemical processes underpinning cancer advancement and creating more effective targeted therapies to intercept these aberrant systems [5,11,12]. This multifaceted nature of cancer progression, recurrence, and therapeutic resistance underscores the need to characterize the ‘interactomes’ of cancer cell surface receptors in an unbiased and comprehensive manner to advance cancer treatment.

Traditional proteomic techniques such as affinity purification and immunoprecipitation often struggle to detect dynamic, transient PPIs occurring within the native cellular environment, particularly those involving membrane-associated receptors. Proximity labeling overcomes many challenges by allowing for the selective labeling, recovery, and identification of proteins that are bound to or near a specific target receptor on the cell surface, including on live cancer cells [13,14,15,16,17]. This approach has become an invaluable tool for mapping the physical interactions of oncoproteins and characterizing the protein microenvironment on cancer cell surfaces. By providing a robust framework for studying tumor cell interactomes, including in heterogeneous tumor contexts, proximity labeling can illuminate receptor-mediated signaling pathways critical to neoplastic processes such as nascent cell transformation, invasive growth, and metastasis [18,19,20,21,22], as well as drug resistance and disease recurrence.

While small-molecule photocatalysts capable of initiating local protein labeling after covalent attachment to an antibody or ligand (referred to as micromapping) have recently been introduced [23,24], as described at length below (Section 3), classical proximity labeling workflows involve expressing a fusion or coupling a promiscuous biotin ligase or peroxidase enzyme to a target protein of interest. Several engineered enzymes have been developed, starting with BioID (Proximity-Dependent Biotin Identification) [18,25,26] and APEX2 (engineered ascorbate peroxidase 2) [27,28], followed by the more advanced TurboID and miniTurboID variants [17,18] engineered to improve labeling efficiency. These enzymes enable covalent tagging of nearby proteins with biotin or reactive substrate handles, such as phenoxyl radicals. Tagged proteins are then selectively isolated using affinity capture resins, like streptavidin beads, and analyzed via tandem mass spectrometry-based proteomics. This process, given the fusion of the proximity labeling probe of choice, does not alter the endogenous function of the target receptor and can generate detailed maps of protein interaction networks and the local molecular microenvironment around the target receptor [18].

Given that the local receptor interaction neighborhoods on cancer cells are of great fundamental and clinical importance, proximity labeling can address key questions in cancer cell biology. By uncovering critical details about a receptor’s local protein microenvironment, proximity labeling can inform the identity of key receptor cofactors, define the biophysical states of receptor complexes, and illuminate downstream signaling mechanisms [29,30]. Such data can provide valuable insights into the functional roles of cancer cell surface receptors, their interaction partners, and their dysregulation during cancer progression, including primary disease, recurrence, and drug resistance [11,12], paving the way for the development of more effective therapeutics. Additionally, this technology holds promise for identifying new biomarkers that can aid in the diagnosis and monitoring of diseases as well as advance the development of multi-target treatments [31,32,33]. A biomarker is a dynamic molecule that indicates an alteration in an individual’s physiological state, relating to health, disease, drug treatment, toxin exposure, or other environmental challenges. Unlike static genetic predispositions such as single nucleotide polymorphisms, biomarkers change over time, reflecting cellular protein expression patterns [34]. The discovery of predictive protein biomarkers is crucial for successful drug discovery and development as they can inform and accelerate decision-making regarding drug efficacy, response, or toxicity based on enhancing sensitivity and/or specificity [35].

In this review, we summarize the historical development, main technical principles, major research applications, specific cancer use cases, and outstanding challenges associated with current prominent proximity labeling platforms, while also outlining promising new directions and the broader utility of this emerging toolkit to advance precision cancer medicine.

## 2. Historical Development of Proximity Labeling Techniques

The evolution of proximity labeling techniques has significantly advanced the field of functional proteomics, enabling more precise analysis of protein interactions within their native organellar compartments, including the cancer cell surface [14,36]. This section highlights major advancements in this family of technologies.

Before the advent of proximity labeling, researchers typically relied on traditional biochemical-based affinity pulldown methods such as affinity purification–mass spectrometry (AP-MS) and immunoprecipitation (IP) to study receptor-mediated interactions in cancer cells. However, these conventional approaches face major limitations in capturing the full complexity of receptor microenvironments. Proximity labeling overcomes these challenges, offering a transformative approach for mapping complex protein networks in their natural cellular context in studying receptor microenvironments in cancer cells.

Traditional methods often miss weak or transient protein interactions that are critical for signaling pathways and may disrupt cellular structures during lysis. Proximity labeling, by labeling proteins in their native cellular context, can capture these interactions, providing a more comprehensive view of the receptor microenvironment in living cells [37,38,39]. Moreover, traditional methods usually capture static snapshots of protein interactions. However, proximity labeling can be performed in live cells, allowing for the capture of dynamic changes in receptor interactions over time [36,40]. This is crucial for understanding the temporal aspects of receptor signaling in cancer progression. Proximity labeling techniques offer high-resolution spatial information about protein interactions by integrating with advanced imaging methods, such as super-resolution microscopy [41]. This integration enables detailed visualizations of receptor interactions on the cancer cell surface, which are challenging to achieve with traditional methods. Proximity labeling is also highly effective for analyzing complex biological samples, like tumor tissues, where traditional methods often face challenges due to the abundance of non-target proteins [20]. This technique is especially valuable for investigating receptor microenvironments in clinical cancer samples. Furthermore, proximity labeling has revealed new biomarkers that traditional methods cannot detect. These biomarkers have the potential to be therapeutic targets or diagnostic indicators, paving the way for personalized cancer treatment options [42,43].

Overall, proximity labeling is ideal for capturing dynamic changes in protein interactions over time, real-time mapping of protein interactions in live cells, and working with complex biological samples, such as tumor tissues. By utilizing these benefits, proximity labeling provides a more comprehensive and precise understanding of receptor microenvironments in cancer cells, surpassing traditional methods such as IP and AP-MS.

An important conceptual forerunner to contemporary proximity labeling is the DamID (DNA adenine methyltransferase identification) DNA-labeling method, pioneered by Steven Henikoff in 2000 [44]. This technique entails coupling a DNA methyltransferase with a chromatin-associated protein, leading to the methylation of nearby bound nucleic acid segments. Researchers can pinpoint genomic areas close to a protein of interest by sequencing methylated sites. DamID established the fundamental idea of employing enzyme fusions to label biomolecules in proximity to a target protein.

In 2004, Choi-Rhee et al. adapted this concept to protein labeling by modifying the endogenous biotin protein ligase BirA from *Escherichia coli* to reduce its strict substrate specificity [45]. By introducing a single mutation (R118G) within the catalytic site, BirA’s binding affinity for the reactive intermediate biotin-5′-AMP was lowered, allowing the enzyme to promiscuously biotinylate nearby protein molecules without requiring a specific amino acid substrate recognition sequence [20].

**Figure 1:**
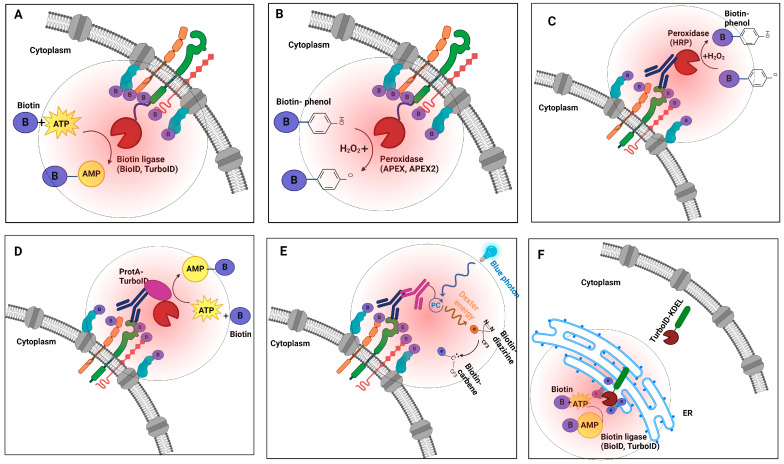
Schematics illustrating the biophysical principles of various proximity labeling strategies for mapping receptor protein interactions on cancer cells. (**A**) Biotin ligase (BioID, TurboID, or miniTurbo) fusion proteins utilize ATP and biotin to catalyze the formation of reactive biotin-5′-AMP, which diffuses and covalently labels proximal proteins. (**B**) Peroxidase-based enzymes (APEX/APEX2) use exogenous hydrogen peroxide to oxidize biotin-phenol into reactive phenoxyl radicals that preferentially label nearby proteins. (**C**) Peroxidase-based strategies facilitate selective labeling of the extracellular protein environment surrounding a specific antibody-bound target. (**D**) Recombinant ProtA-TurboID fusion enables the labeling of proteins in proximity to an antibody-bound target of interest. (**E**) Light-activated labeling: Upon blue light illumination, photocatalyst-conjugated antibodies label receptor proteins on the cell surface with high spatial precision. (**F**) TurboID-based organelle labeling: TurboID allows targeted labeling of localized proteins or trafficking to specific organelles (e.g., ER secretome cargo), enabling detailed mapping of subcellular environments. Collectively, each strategy provides unique capabilities for studying receptor protein interaction dynamics with distinct spatial and functional specificity.

Building on this innovation, Roux et al. [25] adapted the mutated BirA* enzyme as the basis of an innovative in vivo molecular proximity labeling method they introduced as BioID in 2012 (Figure 1A). This method involves expressing the promiscuous biotin ligase (~35 kDa in size) as a fusion to a target protein of interest, which can catalyze the formation of reactive species, leading to covalently tagging of adjacent proteins within a short distance of the fusion in living cells [46]. The biotinylated proteins are then isolated using streptavidin beads and identified via tandem mass spectrometry [15,40].

The original BioID reaction is inefficient, often requiring hours to days to complete, which can result in non-specific labeling. To address this limitation, alternate enzymes, such as APEX (ascorbate peroxidase), were developed to enable efficient labeling reactions in just 1 min [18,47]. In the presence of hydrogen peroxide, APEX produces transient phenoxyl radicals from the exogenous substrate biotin-phenol which then selectively react with electron-rich amino acids, such as tyrosine, that are in close proximity (Figure 1B) [48,49]. This rapid labeling makes these tools well suited for tracking transient PPIs occurring at the cell surface. An improved variant with an A134P mutation that enhances catalytic activity, APEX2, facilitates labeling of the interaction partners of targets expressed at lower cellular levels [28].

A related strategy that maintains native target expression levels and function involves coupling horseradish peroxidase (HRP) to a secondary or primary antibody directed at a protein of interest (Figure 1C). In the presence of H_2_O_2_, the HRP-conjugate catalyzes the localized conversion of a biotin-phenol substrate into short-lived free radicals, resulting in covalent biotinylation of proteins in the immediate vicinity of the target of interest [50,51]. Compared to BioID, APEX/APEX2 offers superior spatial resolution [46,52]. However, careful experimental design and controls are especially critical because this technique involves exposing cells to highly toxic hydrogen peroxide, which can cause cellular stress and introduce potential artifacts (see Box 1).

Box 1 **Considerations when performing enzyme-based proximity labeling.**

 Traditional biochemical approaches like affinity purification can miss weak or unstable interactions, resulting in an incomplete understanding of interaction networks [37,53]. In contrast, enzyme-based proximity methods label proteins irreversibly in a near-native cellular context before cell lysis, allowing for the capture of fleeting binding events, thereby providing a more comprehensive snapshot of target interaction networks [38,39]. Covalent enzyme-based labeling therefore allows one to map transient interactions between cell surface receptors and their transmembrane and intracellular binding partners, which can change rapidly in response to extracellular cues (ligand binding) or post-translational modifications, such as phosphorylation that cause structural changes that cause the recruitment of interaction partners [38,54,55,56]. Overexpression or hyperactivity of labeling enzymes can lead to widespread biotinylation beyond the desired area surrounding a target receptor [52,57]. APEX and TurboID also create reactive species like phenoxyl radicals and other reactive intermediates, which can disperse beyond the intended labeling zone, leading to non-specific biotinylation of proteins located further from the target protein [57]. APEX has also been shown to effectively label RNA in addition to proteins [20] but necessitates the use of hydrogen peroxide, which is toxic to live cells [47]. Conversely, while enzymes offer a gentler approach to studying living samples, high-level expression can contribute to non-specific labeling, including excessive enzyme activity, reactive species diffusion, and endogenous biotinylation [15,42,43,50,58]. For example, when continuously expressed in tissues, TurboID can deplete biotin, resulting in reduced survival and stunted growth [18], while extended labeling periods exceeding 24 h can negatively impact cell proliferation in vitro [18]. To address these concerns, researchers can provide exogenous biotin, limit labeling duration by using inducible enzyme expression systems, or restrict expression to specific cell types in animal models to maintain normal physiological function [18,20]. Directly fusing an enzyme to a target protein can potentially lead to other artifacts. For example, the enzyme may interfere with a receptor’s function, such as ligand binding or downstream signaling, potentially altering its interaction profile [17,59]. The enzyme fusion may also disrupt the proper localization or trafficking of the target protein to its intended subcellular compartment, resulting in the labeling of proteins in incorrect cellular contexts and confounding data interpretation [13,17,60]. To address these limitations, optimizing an enzyme construct by exploring different linker sequences, orientations, or alternative fusion strategies is essential [17,60]. Functional validation tests should also be conducted to independently verify that the resulting fusion protein retains its native function and subcellular localization [58]. The use of recombinant ProtA-TurboID offers a potentially more versatile approach as it is applicable to fixed and non-fixed sample applications and does not require altering genetic manipulation and so can be used to probe primary cells and tissue sections. Its utility relies heavily on the specificity and quality of the primary antibodies employed, which can pose challenges for some targets. For experiments focused on low-abundant targets, low signal-to-background labeling may complicate data analysis [61]. Regardless of the method, there are major practical challenges in performing proximity labeling and preparing samples for mass spectrometry that the authors of this paper have encountered. The target or ligase enzyme fused to the target often needs to be overexpressed in cells to achieve a sufficiently high labeling intensity. To address this issue, cells can be transfected or transduced to boost target levels. Additionally, using an antibody-based proximity labeling platform with ProtA-TurboID is not suitable for cells expressing Fc fragments or various immunoglobulins on their surface as Protein A may interact with these fragments in addition to the antibody-bound target of interest, leading to increased non-specific interactions and reduced labeling efficiency. To mitigate this, alternative proximity labeling platforms, such as photocatalytic-based labeling (see Box 2), can be employed. A common issue with immunoprecipitation/mass spectrometry is non-specific binding by non-biotinylated or biotinylated proteins to streptavidin beads, which can result in the co-purification of false positives. Other strategies can also be used to reduce background and non-specific binders, including optimizing harsh washing steps, reducing protein concentration, and minimizing the number of streptavidin beads to reduce non-specific background binding, increasing the signal-to-noise ratio (Table 1).

**Table 1:** Overview of PL enzymes.

Enzyme	Type	Enzyme Size (kDa)	Labeling Time	Modification Sites	Advantages	Limitations
BioID	Biotin ligase	35	18 h	Lys	Non-toxic for in vivo applications	Poor temporal resolution, low catalytic activity
BioID2	Biotin ligase	27	18 h	Lys	Non-toxic for in vivo applications, higher activity than BioID, and stable at higher temperatures	Poor temporal resolution, low catalytic activity
TurboID	Biotin ligase	35	10 min	Lys	Non-toxic for in vivo applications, highest activity biotin ligase	Potentially less control of labeling window, potential toxicity in long-term experiments
miniTurbo	Biotin ligase	28	10 min	Lys	Non-toxic for in vivo applications, high activity, smaller than TurboID, and high temporal resolution	Lower catalytic activity and stability compared with TurboID
ProtA-TurboID	Biotin ligase	35	10 min	Lys	Be applicable to studying any desired cell type or primary material	Depends on the abundance of the targeted protein
APEX	Peroxidase	28	1 min	Tyr, Trp, Cys, His	High temporal resolution, versatility for both protein and RNA labeling	Limited application in vivo because of the toxicity of H2O2
APEX2	Peroxidase	28	1 min	Tyr, Trp, Cys, His	High temporal resolution, versatility for both protein and RNA labeling	Limited application in vivo because of the toxicity of H2O2
HRP	Peroxidase	44	1 min	Tyr, Trp, Cys, His	High temporal resolution, versatility for both protein and RNA labeling	Limited application in vivo because of the toxicity of H2O2, limited to the secretory pathway and extracellular applications

In a significant technical advance in 2018, Alice Ting’s team introduced two promiscuous biotin ligases, TurboID and miniTurbo. TurboID contains 15 site-specific amino acid substitutions compared to wild-type BirA. In comparison, the smaller but less active 28 kDa variant miniTurbo features 13 substitutions but lacks the inhibitory N-terminal domain of BirA [18]. These variants exhibit up to a >20-fold increase in labeling efficiency in vivo compared to BioID, enabling the labeling of cellular interaction partners within just a few minutes while eliminating the need for toxic chemicals. The quick and efficient labeling capabilities of TurboID and miniTurbo are particularly advantageous for studying dynamic multi-protein complexes such as those present on the cancer cell surface (Figure 1D) [61,62]. Enzyme fusions can be expressed after transient transfection of plasmids into cultured cells or after delivery of viral constructs (e.g., lentivirus) directly into tumors and other tissues, including mouse models.

In a variation of the direct target fusion strategy, a recombinant ProtA-TurboID enzyme has also been developed as an “off the shelf” biotinylation tool analogous to immunofluorescence. Upon engagement with the Protein A moiety, the TurboID domain can be brought in proximity to label around a cellular target that is pre-bound to an antibody (Figure 1E) [61,63]. Santos-Barriopedro et al. used this indirect labeling strategy to probe the proximal proteomes of antibodies targeting three nuclear targets (Emerin, H3K9me3, and BRG1) in both fixed and live cells [61] (Table 1).

Proximity labeling can be quantified using mass spectrometry, which identifies and quantifies biotinylated proteins. For example, proximity labeling coupled with quantitative MS can be achieved using metabolic labeling methods like SILAC (stable isotope labeling by amino acids in cell culture) or in vitro chemical labeling techniques such as iTRAQ (isobaric tags for relative and absolute quantification) and TMT (tandem mass tags) [14]. Maffuid et al. utilized the EXCELL (enzyme-mediated intercellular proximity labeling) method to study immune–tumor cell interactions that demonstrate that proximity labeling can indeed be quantitated. This method uses a modified sortase A enzyme (mgSrtA) expressed on the membrane of tumor cells to label interacting immune cells. This technique allows for the detection and characterization of immune–tumor cell interactions in a time- and concentration-dependent manner, both in vitro and in vivo. The quantitation is achieved by measuring the extent of labeling, which correlates with the proximity and frequency of interactions between immune and tumor cells. This quantitative aspect is crucial for understanding the dynamics of immune cell recruitment and interaction within the tumor microenvironment, providing valuable insights for cancer immunotherapy strategies [38].

## 3. Alternative Proximity Labeling Techniques

By capturing protein interactions in a near-native physiological context, BioID/TurboID effectively addresses many limitations of earlier biochemical techniques, such as difficulty detecting transient or weak interactions. Still, enzyme-based approaches do suffer from certain challenges (as described in Box 1). More generally, unfused proximity labeling enzymes can be expressed throughout an entire cell to label all protein constituents, or they can be targeted using trafficking signals to label specific organelles. For instance, the endoplasmic reticulum (ER) can be targeted to tag proteins destined for secretion or as cell surface receptors that will be inserted into the plasma membrane. The KDEL sequence (Lys-Asp-Glu-Leu) is a commonly used ER retention signal that can be fused to proteins to target them to the ER lumen. KDEL-TurboID fusions enable selective labeling and identification of secretory proteins and receptors transiting the ER lumen = as cargo via the secretory system [64,65] (Figure 1F). Similarly, proximity labeling enzymes can be directed to other subcellular regions of interest, such as compartments connecting adjacent cells (e.g., cell–cell synapses), to preferentially recover protein components present in these particular areas. For example, Li et al. developed a rapid technique for labeling cancer cell surface proteins, known as peroxidase-mediated cell surface labeling (PECSL). This method uses HRP-fused cell surface proteins (including INSR, CTNNB1, TFRC, IGF2R, and SORT1 in this study) to convert aryl azide-biotin or phenol-biotin reagents into active radical species. These radicals rapidly label tyrosine residues on neighboring proteins in just one minute [66]. Likewise, Rhee et al. adapted APEX-mediated proximity labeling to address the shortcomings of traditional mitochondrial purification techniques [67].

Orthogonal non-enzyme-based proximity labeling methods have been reported these past few years [23,24,68,69,70]. In this chemical ‘micromapping’ approach, protein labeling is triggered by visible light-directed (usually blue wavelength) photoactivation of a small-molecule photocatalyst coupled to a primary or secondary antibody, which in turn generates reactive biotin-carbenes from a diazirine substrate such as biotin-tyramide, through a process known as dexter energy transfer. This photo-directed approach allows for tighter spatiotemporal control of protein tagging and higher specificity of the resulting proteomic coverage compared to enzyme-based techniques [71]. The development of innovative photo-labeling techniques enables precise, high-fidelity, high-resolution capture of local receptor protein networks on both cancer cells and other adjacent cells in the tumor microenvironment [23,69,72]. Nevertheless, while photocatalyzed micromapping can be used to define the interaction neighborhoods of native targets expressed at endogenous levels, this strategy depends on access to suitable high-selectivity antibodies or ligands for accessible targets (see Box 2) and current workflows are optimized for abundant cell surface markers.

**Figure 2:**
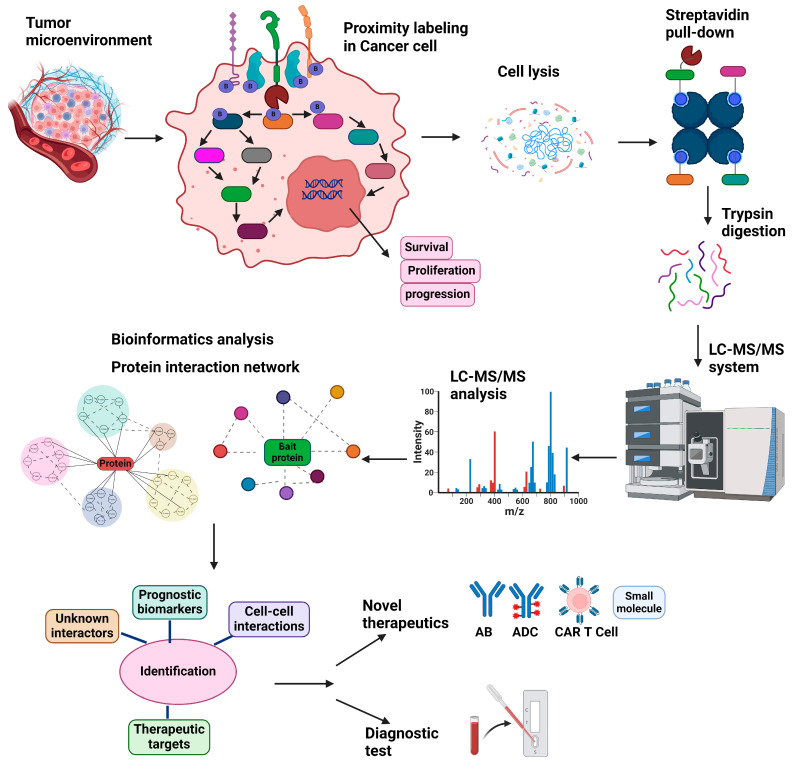
Generalized overview of proximity labeling workflow as applied to mapping receptor protein interactions on cancer cells. The process begins with proximity labeling of cell surface receptors or other targets on cultured cancer cells or heterogeneous tumor samples expressing the protein of interest. Following labeling, the cells are lysed, and biotinylated proteins are isolated using streptavidin beads. The captured proteins are enzymatically digested with trypsin and analyzed using liquid chromatography–tandem mass spectrometry (LC-MS/MS). Data analysis and bioinformatic filtering then generate a scored protein interaction network, revealing potential unknown interaction partners. By characterizing receptor protein neighborhoods on cancer cells, this workflow can reveal fundamental mechanisms driving pathology, identify prognostic biomarkers, and uncover novel therapeutic targets, including for biologics such as antibodies, antibody–drug conjugates, engineered Chimeric Antigen Receptor (CAR) T-cells, and classical small-molecule inhibitors.

Regardless of the approach used, after performing a proximity labeling reaction, the cells are lysed under denaturing conditions, and biotinylated proteins are purified using streptavidin beads or anti-biotin antibodies [73]. The enriched proteins are digested with trypsin to generate peptides, which are then analyzed by mass spectrometry. The peptide ions’ mass-to-charge (*m*/*z*) ratios and fragmentation patterns are used to identify the corresponding proteins. Finally, bioinformatics tools are employed to reconstruct and analyze the protein interaction network, and the results are cross-referenced against curated interaction databases and functional annotation resources (Figure 2).

Box 2 **Considerations for deploying photocatalytic-based labeling.**

Compared to enzyme-based methods, photo-proximity labeling can achieve tighter spatial resolutions ranging from ~50 nm down to a few angstroms depending on the half-life of the reactive substrate used [20,24,46,49,53]. This tight radius can reduce non-specific labeling, ensuring the detection of only proteins directly bound to a target or in the immediate vicinity, enhancing confidence in terms of functional relevance. By allowing the capture of dynamic events at the cell surface, spatiotemporally controlled photo-labeling also provides greater biophysical insights [27,28,55]. This precision is crucial for understanding the complex signaling networks involving cell surface receptors, which regulate dynamic intracellular signaling processes that ultimately drive tumor cell proliferation, migration, and survival [38]. In terms of limitations, the success of photocatalyst-based micromapping is generally limited to abundant cell surface targets with suitable high-selectivity binding reagents and access to specialized equipment for photoactivation. The efficacy of photocatalytic labeling can be affected by several factors, most notably the depth of light penetration. This limitation is particularly significant when working with complex biological samples or dense tissue environments, where light may not reach all target areas uniformly. Despite the stringent purification process afforded by streptavidin enrichment, other challenges remain. Non-specific binding of non-biotinylated proteins to streptavidin beads or biotinylated proteins can lead to co-purification of false positives [46,52]. Although extended and rigorous washing steps can minimize non-specific binding [46], some cells or tissues naturally contain natively biotinylated proteins, which add to the background signal and complicate data interpretation [38,52]. Additionally, non-selective biotinylation of irrelevant but abundant surface proteins can increase background, making it difficult to distinguish genuine interactors from non-specific associations [59]. To mitigate these limitations, several strategies are employed. Optimizing labeling conditions—such as antibody levels, shortening labeling duration, or lowering substrate concentrations—can minimize off-target biotinylation while maintaining effective labeling near the target protein [19,58]. Quantitative proteomics approaches, such as isotope tagging or label-free quantification, that enable ratiometric analysis can also help differentiate specific interactors from the background based on enrichment ratios compared to negative (isotype IgG) controls [14,57]. Additionally, complementary validation experiments, including reciprocal co-immunoprecipitation and imaging-based proximity ligation assays, are essential for verifying interactions prioritized based on novelty and potential relevance to cancer biology [18,57].

## 4. Proximity Labeling Strategies to Study Receptor Signaling Networks in Cancer Cells

One major application of proximity labeling is in mapping receptor microenvironments. Cell surface receptors are integral to signal transduction, where they mediate communication between a tumor cell and its microenvironment, influencing processes such as cell growth, differentiation, and survival. Aberrations in these signaling pathways often lead to uncontrolled cell proliferation, a hallmark of cancer [1,2,74,75,76,77]. The introduction of both enzyme- and photocatalyst-based proximity labeling techniques has marked significant technical and conceptual steps forward in studying receptor interaction neighborhoods in cancer cells. Since proximity labeling methods enable the labeling of interacting proteins within live cells, they preserve the native context and avoid disruptions caused by cell lysis or protein extraction [54,56]. This preservation is crucial for studying cancer cell surface receptors, which are involved in intricate signaling pathways that involve dynamic interactions following receptor ligand or drug engagement that lead to post-translational modifications and spatial reorganization [38].

Enzyme-based proximity labeling has also been used to map interactions of receptor protein kinases. For instance, Liu et al. used TurboID to study the interactome of the FLT3 receptor tyrosine protein kinase, which is frequently mutated in acute myeloid leukemia (AML). In addition to wildtype Flt3-TurboID, they assessed FLT3-ITD (Internal Tandem Duplication of a juxtamembrane motif that is constitutively activated in the absence of FLT3 ligand) and the FLT3-TKD variant (Tyrosine Kinase Domain with point mutations in the kinase activation loop leading to constitutive receptor activation) that show impaired regulation of kinase activity. The authors utilized Ba/F3, MV4-11, and RS4-11 cell lines in their study. They identified a novel association between FLT3-ITD and BRCC36, a specific K63-linked polyubiquitin deubiquitinase, not observed with wild-type FLT3 or FLT3-TKD, suggesting a new mechanism by which FLT3-ITD stability and oncogenic signaling are enhanced to promote uncontrolled cell proliferation [78]. Petersen et al. employed TurboID to reveal the interactome of Abelson interactor 1 (ABI1) and its role in cancer development. In this study, researchers successfully identified 212 ABI1 proximal interactors and revealed previously unknown associations between ABI1 and proteins involved in the TAK1-IKK pathway, including TAK1, TAB2, and RIPK1 in NIH/3T3 cell line. They also uncovered a new function of ABI1 in TAK1-NF-κB inflammatory signaling, which was further validated through functional assays [79].

Geng et al. employed BioID2 proximity labeling to investigate the functional interactome of DRAM1 in NCI-H1975 and PC9 and A549 non-small cell lung carcinoma (NSCLC) cells. By fusing BioID2 to DRAM1, they successfully identified key interacting partners contributing to DRAM1’s tumor suppressor role. This approach uncovered EPS15 as a crucial interactor promoting EGFR endocytosis, and the V-ATP6V1 subunit as another interactor enhancing EGFR lysosomal degradation. These findings revealed a novel mechanism by which DRAM1 suppresses NSCLC oncogenesis through the regulation of EGFR trafficking and degradation [80]. Nieto et al. fused the BioID2 biotin ligase to programmed death-ligand 1 (PD-L1) and they identified novel interacting partners that contribute to both immune suppression and cancer progression. This study successfully uncovered cancer cell-intrinsic signaling mechanisms initiated by PD-L1, including interactions with ILF2 and ILF3, which activate STAT3 signaling in CUHN013 and CUHN036 cell lines [81].

The APEX enzyme technology has also been used to map interactions of targets on the cell surface. For example, Zhen et al. created an APEX2-FGF1 fusion protein to map the microenvironment of fibroblast growth factor 1 (FGF1) at the cell surface, they uncovered previously unknown interactions between FGF1 and the proteoglycans CD44 and CSPG4, presumably missed by traditional methods, thereby expanding its signaling network [82].

Likewise, Rees et al. developed a conceptually similar approach for mapping cancer cell surface interactors called selective proteomic proximity labeling using tyramide (SPPLAT), which also employs HRP-mediated labeling. In this method, peroxidase is targeted via a specific antibody to the plasma membrane protein of interest and a biotin-tyramide derivative is briefly added. The peroxidase generates a biotin-tyramide free radical that covalently biotinylates proteins in close proximity to the target. They used this platform to identify neighbors of the immune checkpoint protein B7-H4 on the surface of an SKBR-3 breast cancer cell line [83]. In a similar vein, Seo et al. employed HRP-conjugated anti-CD147 antibodies to identify proteins proximal to CD147 on breast cancer stem cells, which revealed an interaction between CD147 and CD276 within lipid raft microdomains that is essential for cancer cell stemness and chemoresistance [84]. 

Enzyme-based proximity labeling has also proven to be a particularly valuable tool for unraveling oncogenic signaling pathways. For example, Kovalski et al. employed BioID proximity labeling to investigate the functional interactome of oncogenic Ras proteins in cancer cells. By fusing BirA* to wild-type and mutant Ras isoforms, they identified both known and novel Ras-interacting proteins. This approach revealed mTORC2 as a previously unrecognized Ras effector [85]. BioID2 has also been fused to the N-terminus of p38α to map multiple upstream and downstream factors in the p38 MAP kinase cascade such as MKK3, MAPKAPK2, TAB2, and c-jun in HEK293T cells [86]. These findings significantly advance the understanding of aberrant signaling in tumors and highlight the potential to discover new therapeutic targets.

Li et al. developed the successful application of antigen–antibody proximity labeling (AAPL) to map protein interactions on cancer cell membranes. This application involves modifying antibodies with a catalytic probe that induces oxidation when the antibody binds to its target antigen. This oxidation marks nearby proteins, allowing researchers to identify proteins interacting with the target antigen. By modifying antibodies with an Fe (III) catalytic probe, the researchers were successfully able to achieve precise labeling of proteins in close proximity to the target antigen. This method was demonstrated using antibodies specific to sodium/potassium transporting ATPase (ATP1A1) and epidermal growth factor receptor 2 (ERBB2), allowing for the identification of unique protein interactions associated with these targets in cell lines PNT2 and SKBR3, respectively. Moreover, the application of AAPL was applied to map interactors of liver–intestine cadherin (CDH17) in colon cancer cells [51].

## 5. Precise Photocatalyst-Directed Micromapping of Surface Receptor Neighborhoods

Photocatalytic proximity labeling, colloquially known as microenvironment mapping or micromapping, is a rapidly emerging antibody-based labeling approach that employs photocatalyst-conjugated antibodies to precisely label tyrosine residues on nearby proteins located close to the target on the cell surface [24,87]. In this labeling scheme, photocatalysts are first attached covalently to an antibody directed to a protein of interest. The first implementation of this technique used iridium (Ir) photocatalysts that, when exposed to blue light, transform diazirines into highly reactive carbenes with an extremely short half-life (about 1 nanosecond) due to quick neutralization by water. These properties result in precise labeling within a radius of ~50 nm of the target, allowing for high-resolution mapping of interaction interfaces [23,24,41,88]. MacMillan et al. introduced a novel platform called μMap (MicroMapping) that employs photocatalytic proximity labeling for mapping protein microenvironments on cell surfaces with high spatiotemporal resolution. This approach offers higher precision in mapping microenvironments compared to enzymatic proximity labeling platforms. The μMap technique successfully identified the constituent proteins of the programmed death-ligand 1 (PD-L1) microenvironment in live lymphocytes and demonstrated the ability to map protein interactions within the immunological synapse between T-cells and antigen-presenting cells. This method showed potential for both cis-labeling (on the same cell surface) and trans-labeling (across cell–cell junctions) [70].

Photocatalytic proximity labeling has subsequently been utilized for high-resolution mapping of other receptor protein microenvironments on cancer cell surfaces, including that of EGFR, c-MET [68,88], and HER2 [23]. Oslund et al. introduced a related approach called photocatalytic cell tagging (PhoTag) for interrogating cell–cell interfaces, particularly interactions occurring at an immune cell synapse. This method employs single-domain antibodies, or else bispecific nanobodies, conjugated to photoactivatable flavin-based cofactors. Upon irradiation with visible light, the flavin photocatalyst generates phenoxy radical tags, enabling the biotinylation of proteins in close proximity to the nanobody targets. The efficacy of PhoTag was demonstrated by selectively labeling synaptic proteins across the PD-1 receptor/PD-L1 ligand axis, bridging antigen-presenting cells with T-cells [69]. Photocatalytic proximity labeling can also be utilized to identify interactions between host cells and various pathogens by integrating photocatalysts directly into viral or bacterial components. Datta et al. used µMap to probe interactions between the SARS-CoV-2 spike protein and ACE2-expressing HEK293T cells [89].

By analyzing protein interactions and the molecular surroundings of specific cancer-associated receptors, researchers can use photoproximity labeling probes to document changes in receptor complexes during cancer progression and reveal distinct differences in receptor interaction neighborhoods in normal, pre-cancerous, and malignant cells.

## 6. Illuminating Pathways Driving Disease Progression

Receptors also play a role in modifying the extracellular matrix, promoting angiogenesis, and evading immune surveillance, which are critical steps in cancer progression [75,90,91,92,93,94]. During metastasis, cancer cells detach from the primary tumor, invade surrounding tissues, and establish secondary tumors in distant organs. Cell surface receptors mediate these processes by facilitating cell adhesion, migration, and invasion, making them potential targets for therapeutic intervention [33,95,96,97,98]. Receptors can also serve as biomarkers for cancer diagnosis and prognosis. Their expression levels can provide insights into the aggressiveness of the tumor and its potential response to treatment, aiding in personalized medicine approaches [99,100,101,102]. Furthermore, cell surface proteins, such as drug efflux pumps, can contribute to drug resistance. By understanding their mechanisms, new strategies can be developed to overcome resistance and improve the efficacy of existing treatments [101,103,104,105]. 

Proximity labeling approaches are transforming the study of protein interactions and local proteomes in cell culture models, such as cancer cell lines. These methods also facilitate the identification of receptor-mediated protein interactions within their local plasma membrane microenvironments, offering insights that traditional proteomics techniques often cannot achieve [106]. By analyzing protein interactions and the molecular surroundings of specific cancer-associated receptors, researchers have uncovered new binding partners, documented changes in receptor complexes during cancer progression, and revealed distinct differences between receptor environments in normal and cancerous cells. These findings have significantly advanced our understanding of aberrant receptor signaling in tumors and highlighted potential new therapeutic targets and approaches [20,23,68,70,88,107,108].

## 7. Applications for Proximity Labeling to Study Cancer Progression

Proximity labeling is also a powerful technique that can transform cancer research by enabling detailed exploration of tumor cell biology and the intricate interactions within the complex tumor microenvironment. Here, in the absence of robust unbiased single-cell proteomics, cell labeling is particularly valuable for mapping the source of intrinsic signaling effector proteins secreted by tumor cells into the complex molecular environment mediating cell–cell interactions despite multiple other confounding cell types. The technique can also be used to explore how cancer cells interact with surrounding stromal cells, immune cells, and the extracellular matrix, leading to immune bypass and resistance to therapy. For instance, studies have revealed the interactions between cancer cells and tumor-associated fibroblasts or immune cells, uncovering potential strategies to disrupt these supportive networks and inhibit tumor progression [31,69,70,88].

For example, Yang et al. developed a genetically engineered mouse model for in vivo proximity labeling of the mammalian secretome. They created a secretome reporter strain by inserting DNA sequences encoding an endoplasmic reticulum-directed promiscuous biotin ligase, BirA*G3, into the ubiquitously expressed Rosa26 locus. This BirA*G3 mouse model had improved labeling efficiency and tissue specificity compared to viral transduction. By enabling enzyme-catalyzed proximity labeling of secreted proteins in live animals [106], this approach allows for sensitive detection of endogenous secretomes across tissues. When applied to tumors, this approach addresses previous challenges in identifying low-abundance secreted proteins and tracking protein interactions between different cells of origin in vivo. By providing critical cell-type resolved functional insights that are essential for understanding cancer cell crosstalk within the tumor milieu, such as between cancer cells and immune cells leading to immune suppression, more effective therapeutic strategies could potentially be devised.

Proximity labeling can be used to monitor changes in receptor–protein interactions after drug treatment, offering insights into how the drug mechanism of action and resistance develops. This approach enables observation of dynamic changes in PPIs over time, revealing how signaling networks evolve in response to therapy. By comparing receptor interactions in drug-sensitive versus drug-resistant cells, researchers can identify protein associations contributing to refractory behavior. Furthermore, proximity labeling can uncover new protein interactions that arise as cancer cells adapt to a drug, potentially highlighting alternative signaling pathways that circumvent the drug’s target [12,50].

**Figure 3:**
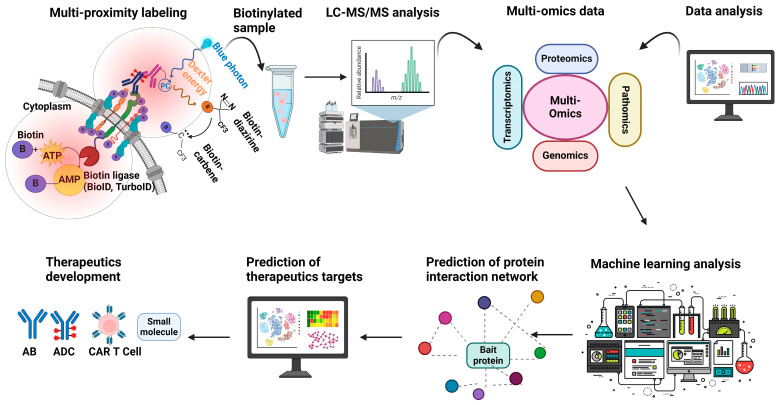
Utility of proximity labeling for the development of therapeutics for cancer. Proximity labeling is a powerful tool for developing new cancer treatments by enabling precise mapping of receptor-associated protein interactomes at spatial scales to improve understanding of drug mechanisms of action or to discover potentially actionable new targets. Enzyme-based methods target intracellular receptor domains, while antibody-based photocatalytic techniques label extracellular regions; antibody–drug conjugates (ADCs) can also define target functionality and specificity. Mass spectrometry data from labeled samples can then be integrated with other datasets, such as spatial proteomics or genomics, transcriptomics, and multiplex imaging, using machine learning algorithms to construct predictive models and identify markers of clinical response and targets in the cancer receptor microenvironment. This comprehensive approach can inform the development of innovative therapies, including biologics, bispecific antibodies, ADCs, and small-molecule inhibitors, advancing precision medicine to improve cancer treatment.

Another important use of proximity labeling in cancer biology is the identification of novel therapeutic targets and biomarkers (Figure 3). The technology allows researchers to map the proteomes of specific subcellular compartments and protein complexes, identifying unique interactions that occur only in cancer cells. This can lead to the discovery of new drug targets and biomarkers for diagnosis or prognosis [68,69,108]. For example, proximity labeling has been employed to study dimerized surface receptors such as HER2 in breast cancer [42] and FLT3 in AML [43,109]. These receptors are critical drivers of cancer progression due to their roles in activating signaling pathways that promote abnormal cell growth, survival, and metastasis.

Cancer cell-specific receptors also serve as crucial targets for developing targeted therapies, including bispecific antibodies (biologics) that show enhanced tumor cell selectivity. Targeted therapies aim to bind and inhibit receptors and their downstream signaling pathways, or internalize payloads (antibody–drug conjugates or ADCs), thereby reducing the growth and spread of cancer cells while minimizing unintentional damage to normal cells [110,111,112,113,114]. By investigating proteins closely associated with the dimerized forms of certain receptors, researchers have uncovered specific molecules that could serve as therapeutic targets or provide deeper insights into the mechanisms of cancer development [23,24,68,78]. CAR T-cell therapy is another prominent example, utilizing receptors for the recognition and elimination of cancer cells via engineering T-cells expressing engineered chimeric receptors that recognize specific surface antigens on tumor cells, thereby enhancing the immune system’s ability to target and destroy malignant cells [95,115,116]. Advances in both virtual and experimental screening technology have enabled the rapid production of antibody-based reagents targeting cell surface receptors. These therapies can be designed to recognize combinations of specific receptors preferentially on cancer cells, minimizing damage to normal tissues and improving therapeutic outcomes [117,118,119,120]. Conversely, the ADC strategy involves targeting and uptake by cancer cells of toxins attached to receptor-binding antibodies. This approach leverages the high expression of certain receptors on cancer cells to deliver toxic payloads selectively, but the events following endocytosis, internalization, and release of the cytotoxic agent are less well understood, so the application of proximity labeling could potentially navigate optimal delivery routes while mitigating the challenge of receptor expression on normal tissues (Figure 3) [1,42,121,122,123].

Proximity labeling can also be applied to clinical diagnostics or treatment optimization by discovering and validating new biomarkers. For instance, cell surface proteins identified through proximity labeling of proteins secreted by pancreatic cancers could potentially be developed into a blood test for early detection. Similarly, proteins that interact with a drug target only in responsive cells could help predict which patients will benefit from a particular therapy.

A rigorous validation process is crucial to ensure that biomarkers discovered through proximity labeling are thoroughly tested before clinical use. For example, a protein found to interact with an oncogenic receptor in drug-resistant cancer cells could be verified as a specific marker of treatment resistance, potentially improving patient stratification and treatment selection [124].

## 8. Conclusions: Challenges, Emerging Trends, and Future Directions

Over the past two decades, proximity labeling has evolved into a cornerstone technique for mapping protein interactomes in cells and tissues to uncover key cofactors of important proteins across cellular compartments, from the cell surface to interior organelles such as the nucleus. This transformative suite of powerful techniques can be tailored to address specific experimental goals, each with unique advantages and limitations. Techniques like the foundational BioID and versatile TurboID enzyme platforms to the more cutting-edge photo-proximity labeling techniques are revolutionizing the study of cancer cell biology, particularly the dynamic macromolecular landscape underlying receptor neighborhoods, oncoprotein signaling, and tumor microenvironments in increasingly sophisticated models.

This review has chronicled the evolution, principles, strategies, and technical considerations associated with the most prominent proximity labeling approaches, emphasizing their growing importance for probing cancer biology. Proximity labeling has already proven especially valuable for studying cancer cell surface receptors in their native states within living multi-cellular tumor systems, circumventing the artifacts and limitations of traditional biochemical pulldown techniques. Enzyme-based methods can target intracellular receptor subnetworks, while antibody-based techniques can be exploited to capture extracellular interaction scaffolds. This complementary dual labeling capability provides a more holistic view of receptor interactions and dynamics, combined with the ability to perform cell state-specific proximity labeling, which will enhance understanding of receptor crosstalk and intercellular signaling occurring within multi-cellular tumor contexts.

Unlike single-cell proteomics, which aims to capture a snapshot of protein expression in individual cells, proximity labeling provides a detailed view of specific protein neighborhoods and their local interactions. While single-cell proteomics can highlight cellular diversity within tumors, proximity labeling can reveal the functional relationships between proteins, potentially identifying new signaling components or regulatory mechanisms in cancer cells [125]. While spatial omics maintains the spatial context of protein expression within tissues, providing insights into the tumor microenvironment and cellular interaction landscape, it does not directly capture protein-level interactions. Proximity labeling therefore complements spatial profiling by supplying protein-level interactome data that can potentially be mapped onto spatial contexts, giving a more comprehensive view of cellular function. For example, proximity labeling can show how protein interactions vary across different regions of a tumor, such as the core versus the leading edge, which spatial transcriptomics alone cannot reveal [126,127].

In parallel with spatial proteomics, proximity labeling on pathology tissue sections could enable the creation of high-resolution maps to define the cell surfaceomes of distinct cell states in heterogeneous human tumor biopsies. These maps could also identify functional markers at the interface of different cell types within a tumor and their underlying biomolecular interactions that could potentially be exploited to create combination therapies that counter immune tolerance and metastatic dissemination. Combining these techniques holds great potential for understanding the complex interplay between protein expression, protein interactions, and spatial organization in cancer biology, potentially leading to new therapeutic strategies and biomarker discoveries.

By offering unparalleled resolution and biophysical insights into cancer cell receptor expression, effector protein secretion, and signaling pathway activity, proximity labeling is also poised to become a pivotal framework for understanding the mechanisms driving cancer progression. It illuminates tumor-intrinsic pathways governing growth, proliferation, and survival while revealing key aspects of cell–cell crosstalk that contribute to immune evasion, drug resistance, and disease recurrence. For example, researchers can leverage single-cell expression data to design cell state-specific promoter-driven TurboID reporters to selectively label proteomic networks of transient cancer cell subpopulations during tumor progression from indolent growth to malignant transformation, followed by metastatic dissemination and niche colonization in animal models, providing refined spatial and functional insights that inform precision diagnostics and therapeutics.

In the near future, proximity labeling could be integrated with bispecific antibodies and antibody–drug conjugates (ADCs) to enhance understanding of cancer immunobiology, investigate drug mechanisms of action after target engagement and intracellular uptake, and accelerate the development of multi-targeted therapies. Furthermore, the simultaneous application of diverse proximity labeling methods can enhance comprehensive mapping around both the extracellular and intracellular domains of receptors to provide a more complete picture of receptor interactions and dynamics. For instance, enzyme-based labeling could be employed to target the intracellular domain of a receptor, while antibody-based labeling could simultaneously capture the extracellular domain. Furthermore, multiple receptors could be concurrently labeled and analyzed using different PL methods, enabling a more holistic understanding of complex cellular signaling networks (Figure 3).

The integration of proximity labeling with automated structural modeling tools like AlphaFold offers insights into cancer protein interaction networks at the atomic level. This powerful combination would enable a detailed mechanistic understanding of structure–function relationships, facilitating the design of interface-specific small molecules, molecular glues, and bifunctional proteolysis-targeting chimeras (PROTACs) for targeted protein degradation, as well as high-affinity antibodies. AlphaFold bimolecular predictions complement proximity labeling by confirming and refining identified PPIs, allowing for the identification of potential interaction interfaces for further experimental validation. For instance, a recent study utilized AlphaFold-Multimer predictions to independently validate over 20 neighbors of the epidermal growth factor receptor (EGFR) identified through proximity labeling, generating credible binary interaction models. This synergistic approach accelerates the development of precision cancer therapies by providing a more comprehensive understanding of protein interactions and their structural basis. In parallel, machine learning algorithms could be used to integrate the results of proximity labeling experiments with datasets arising from genomics, transcriptomics, and multiplex imaging (pathomics) studies of human pathology to elucidate how specific interactions and macromolecular assemblies contribute to drug sensitivity, therapeutic resistance, and patient outcomes (Figure 3).

Despite this immense promise, using proximity labeling on heterogeneous tumor samples complicates the isolation of specific cell types for detailed analysis. This difficulty stems from the varied cellular composition of tumors, which can obscure the identification and study of individual cells. To address this challenge, single-cell transcriptomics technology can be utilized. This cutting-edge technique enables the examination of gene expression at the single-cell level, offering a detailed view of tumor heterogeneity. By employing single-cell transcriptomics, different cell populations within tumors can be identified and characterized, enhancing the understanding of tumor biology and potentially leading to more targeted therapeutic approaches. Moreover, traditional proximity labeling methods often require genetic manipulation, which is not feasible in patient-derived tissues. This limitation poses significant challenges for researchers aiming to study these tissues in their native state. Alternative approaches such as antibody-based proximity labeling or photocatalytic-based proximity labeling offer promising solutions. These methods enable target-specific labeling without the need for genetic manipulation, making them suitable for use in patient-derived samples.

In conclusion, by providing deeper insights into receptor dynamics, tumor microenvironments, and therapeutic vulnerabilities, further advances in proximity labeling are poised to advance cancer research in the coming years, paving the way for a better fundamental understanding of disease processes leading to more effective treatments that ultimately improve cancer as we know it.
